# Antioxidant Rich Extracts of *Terminalia ferdinandiana* Inhibit the Growth of Foodborne Bacteria

**DOI:** 10.3390/foods8080281

**Published:** 2019-07-24

**Authors:** Saleha Akter, Michael E. Netzel, Ujang Tinggi, Simone A. Osborne, Mary T. Fletcher, Yasmina Sultanbawa

**Affiliations:** 1 Queensland Alliance for Agriculture and Food Innovation (QAAFI), The University of Queensland, Health and Food Sciences Precinct, 39 Kessels Rd, Coopers Plains, QLD 4108, Australia; 2 Queensland Health Forensic and Scientific Services, 39 Kessels Rd, Coopers Plains, QLD 4108, Australia; 3 CSIRO Agriculture and Food, 306 Carmody Road, St Lucia, QLD 4067, Australia

**Keywords:** Kakadu plum, *Terminalia ferdinandiana*, antioxidants, antimicrobial activity, food preservation, phytochemicals, polyphenols

## Abstract

*Terminalia ferdinandiana* (Kakadu plum) is a native Australian plant containing phytochemicals with antioxidant capacity. In the search for alternatives to synthetic preservatives, antioxidants from plants and herbs are increasingly being investigated for the preservation of food. In this study, extracts were prepared from *Terminalia ferdinandiana* fruit, leaves, seedcoats, and bark using different solvents. Hydrolysable and condensed tannin contents in the extracts were determined, as well as antioxidant capacity, by measuring the total phenolic content (TPC) and free radical scavenging activity using the 2, 2-diphenyl-1-picrylhydrazyl (DPPH) assay. Total phenolic content was higher in the fruits and barks with methanol extracts, containing the highest TPC, hydrolysable tannins, and DPPH-free radical scavenging capacity (12.2 ± 2.8 g/100 g dry weight (DW), 55 ± 2 mg/100 g DW, and 93% respectively). Saponins and condensed tannins were highest in bark extracts (7.0 ± 0.2 and 6.5 ± 0.7 g/100 g DW). The antimicrobial activity of extracts from fruit and leaves showed larger zones of inhibition, compared to seedcoats and barks, against the foodborne bacteria *Listeria monocytogenes*, *Bacillus cereus,* Methicillin resistant *Staphylococcus aureus*, and clinical isolates of *Pseudomonas aeruginosa*. The minimum inhibitory concentration and minimum bactericidal concentration in response to the different extracts ranged from 1.0 to 3.0 mg/mL. Scanning electron microscopy images of the treated bacteria showed morphological changes, leading to cell death. These results suggest that antioxidant rich extracts of *Terminalia ferdinandiana* fruits and leaves have potential applications as natural antimicrobials in food preservation.

## 1. Introduction

Antioxidants from plants and herbs are progressively being used as alternatives to synthetic antioxidants (like butylated hydroxyanisole, butylated hydroxytoluene, and propyl gallate) to preserve food [1]. The use of synthetic antioxidants is tightly regulated due to the health risks such as potential organ toxicity and carcinogenicity associated with overuse [2]. In addition to being used as natural food preservatives, the use of plant antioxidants as functional food ingredients and/or supplements is also growing, based on new findings regarding their potential biological activities [3]. In particular, plant phytochemicals have received substantial research attention based on their ability to act as reducing agents, hydrogen donors, and singlet and triplet oxygen quenchers [4]. Food safety is an important international concern, as food spoilage, due to bacteria and fungi, causes considerable economic loss worldwide [5]. Due to recent outbreaks of emerging pathogens, such as *Listeria monocytogenes*, and rising worldwide impacts of foodborne illness, consumer concerns over food safety and food formulation have increased [1,5], along with the demand for non-toxic natural food preservatives [5]. Many plant extracts possess antimicrobial activity, however inherent variations in bioactivity and concentration places some limitations on the use of plant extracts in food products [6]. However, plant phytochemicals, such as polyphenols, alkaloids, and polypeptides, are known to retain the microbiological and chemical quality of fresh and processed foods [7].

**Figure 1:**
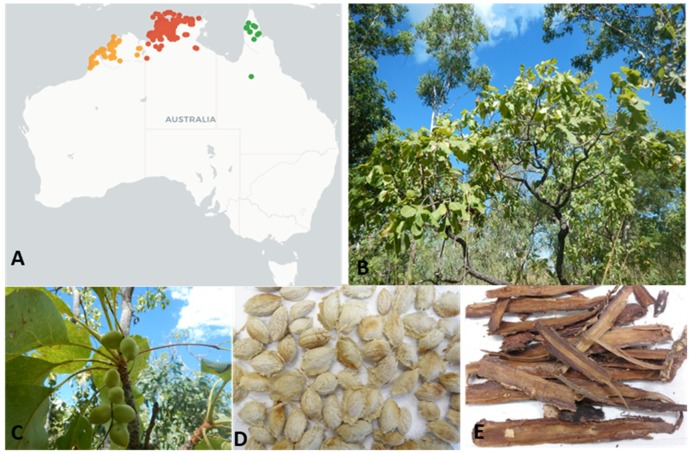
(**A**) Distribution of *Terminalia ferdinandiana* from the Australasian Virtual Herbarium website. (https://avh.ala.org.au) showing ● West Australian, ● Northern Territory and ● Queensland locations. (**B**) Mature tree; (**C**) leaves and fruits; (**D**) seeds; and (**E**) bark.

*Terminalia ferdinandiana* Exell., commonly called Kakadu plum, billy goat plum, gubinge, or salty plum, is a native flowering Australian plant from the Combretaceae family [8]. This endemic Australian, semi-deciduous plant grows in the tropical rangelands of the Northern territory, the Kimberley area of Western Australia, and in some northern parts of Queensland (Figure 1) [9,10]. The fruits are smooth-skinned, fleshy ovoid drupes with a short beak that become yellow-green when ripe (Figure 1). There are about 250 species of the genus *Terminalia* from the family Combretaceae growing in tropical regions across the globe [11]. Among them, approximately 30 species or subspecies of *Terminalia* are endemic to Australia [9,10]. A high degree of phytochemical variability exists amongst different species and subspecies due to genetic diversity, soil and climate conditions, fruit ripening stage, storage, and other post-harvest conditions [12]. 

Due to increasing commercial demand from local and international food industries, *T. ferdinandiana* products are often stored for long periods. Subsequently, the bioactivity and safety of these products must be ensured following long-term storage. A study by Sultanbawa et al. [13] investigated the safe storage of *T. ferdinandiana* extracts and not only confirmed the retention of bioactivity over a period of 18 months in frozen storage (−20 °C), but also identified chemical markers for determining the end-of-storage life of *T. ferdinandiana* extracts. 

Studies focused on the bioactivities of the various phytochemicals in *T. ferdinandiana* fruit and leaves have identified ellagic acid, gallic acid, ethyl gallate, chebulic acid, corilagin, hydroxycinnamic acid, ascorbic acid, α-tocopherol, lutein, tannins, chebulagic acid, exifone, punicalin, castalagin, appanone A-7 methyl ether, xanthotoxin, and phthalane [14,15,16]. The objective of the present study was to determine the potential of *T. ferdinandiana* extracts as antioxidants and antimicrobial agents in food preservation.

## 2. Materials and Methods 

### 2.1. Chemicals

Methanol anhydrous (99.8%), ethyl alcohol (pure), acetone (HPLC grade ≥99.9%), n-hexane (99%), Folin–Ciocalteu’s phenol reagent, gallic acid monohydrate (American Chemical Society (ACS) reagent), 2, 2-Diphenyl-1-picrylhydrazyl (DPPH), n-butanol anhydrous (99.8%), potassium phosphate tribasic (regent grade ≥98%), vanillin, sulfuric acid (99.9%), saponin from Quillaja bark (sapogenin content ≥10%), potassium iodate (99.7–100.4%), tannic acid, and catechin analytical standard were obtained from Sigma-Aldrich (Castle Hill, New South Wales, Australia). Sodium carbonate anhydrous was obtained from Chem-supply, Bedford St, Gillman, South Australia, AU; chloroform (high purity solvent) and trolox were obtained from Merck KGaA, Darmstadt, Germany; HCl (Trace metal grade) was obtained from Fisher Scientific, United States Fisher HealthCare, Veterans Memorial Dr. Houston, Texas, USA. Standard plate count agar (American Public Health Association) (PCA) (CM0463), potato dextrose agar (PDA) (CM 0139), nutrient broth (CM 001), and tryptone soya yeast extract broth (TYSEB) (CM 129B) were purchased from Oxoid Ltd, Basingstoke, UK. Grade AA (6 mm) discs were purchased from GE Healthcare Life Sciences, Whatman, UK.

### 2.2. Sample Collection and Processing

Ripe and mature fruits of *T. ferdinandiana* (total harvest of 5000 kg) were collected from over 600 trees, from native bushland covering a land area of 20,000 km^2^ in the Northern Territory, Australia, in 2015. A voucher specimen, AQ522453, was deposited at the Queensland Herbarium. A portion of the collected fruits were processed by Sunshine Tropical Fruit Products, Nambour, Queensland, Australia, to provide a seedless puree, along with the separated seeds, which were stored at −80 °C until further analysis. The puree was then freeze-dried and milled to provide a uniform powder that was stored at −20 °C and used throughout this study. The freeze-dried puree will be referred to as fruits/fruit extract. The frozen seeds were thawed and washed several times with double distilled water to remove the pulp residue. The seeds were then oven-dried for 48 h at 40 °C. After drying, the seeds were individually cracked using an Engineers vice size 125 (DAWN tools and Vices Pty Ltd, Heidelberg West, Victoria, Australia) to release the kernels from the seedcoats. The seedcoats were processed and analyzed separately in a previous study [17]. The separated seedcoats were hammer milled and used for this study. Leaves and bark were also collected from the same region during the same fruit harvest and were freeze-dried and milled. The milled freeze-dried powders of leaves and bark were used throughout this study.

### 2.3. Preparation of Kakadu Plum Extracts

Accelerated solvent extraction (ASE) (Dionex ASE 200 system, Dionex Corp., Sunnyvale, CA, USA) was performed to prepare the extracts for antioxidant and antimicrobial assays [18]. Briefly, 10 mL stainless steel extraction cells were assembled and fitted with a 27 mm cell filter at the bottom end. Aliquots (1.0 g) of freeze-dried powders of fruits and leaves, and dried powders of seedcoats of *T. ferdinandiana* were mixed with diatomaceous earth (approximately four to five times the weight of the powders to fill the cells completely) and placed in the cells. Five different solvents were used: methanol, ethanol, acetone, hexane, and distilled water. The ASE unit was operated under the following conditions: 60 °C for methanol and ethanol, 50 °C for acetone and hexane, and 75 °C for distilled water; preheat 5 min; static time 5 min, eight extraction cycles, rinse volume 25% with fresh extraction solvent; and purged with 150 psi for 60 sec. The cells containing the samples were prefilled with the respective extraction solvent, pressurized, and heated with the extracts collected into 60 mL amber glass vials. MiVac sample concentrator (GeneVac Inc., New York, NY, USA) was used to concentrate and dry the extracts. The temperatures for solvent evaporation were controlled as follows: methanol and ethanol extracts at 50 °C, acetone and hexane extracts at 45 °C, and water extracts at 70 °C. The concentrated extracts were weighed and stored at −20 °C until analysis.

### 2.4. Antioxidant Capacity

#### 2.4.1. Total Phenolic Content 

The total phenolic content (TPC) of the various solvent extracts of *T. ferdinandiana* tissues was determined by spectrometry using the Folin–Ciocalteu reagent [19]. The extracts (25 µL) were added to the 96-well plate and 125 µL freshly prepared Folin–Ciocalteu reagent and 125 µL sodium carbonate (7.5% *w*/*v*) were also added to the wells. The mixture was incubated in the dark for 30 min at room temperature. Absorbance was measured at 750 nm using a Tecan Microplate Reader (Tecan Infinite M200, Tecan Trading AG, Mannedorf, Switzerland) with Magellan Software (version 6.4, Tecan Trading AG). Results were expressed as g gallic acid equivalents (GAE)/100 g dry weight (DW).

#### 2.4.2. DPPH Radical Scavenging Activity

The DPPH radical scavenging activity assay was performed as per the previously described method [19]. Methanol (200 µL) was used as blank. Control wells contained 100 µL of methanol and 100 µL of DPPH (0.15 mM). Samples (100 µL) and 100 µL of DPPH (0.15 mM) were added to the appropriate wells. The plates were shaken for 15 sec and incubated for 40 min at room temperature and were kept in the dark. Trolox standards at different concentrations (5–35 µM/L) were treated as samples. Absorbance was measured at 517 nm using a Tecan Microplate Reader with the percentage radical scavenging activity of each extract calculated from the standard curve, using the formula described previously [20]; Percentage of radical scavenging activity = (Control_Abs_-Sample_Abs_ / Control_Abs_) × 100%.

### 2.5. Determination of Total Saponin Content

Saponin extracts were prepared as previously described by Xi, et al. [21], with modifications. Briefly, 0.5 g powdered *T. ferdinandiana* fruits, leaves, seedcoats, and barks were extracted three times with 80% ethanol at a ratio of 1:10 *w*/*v* under reflux at 80 °C for 1 h. The combined alcohol extract was concentrated, suspended in distilled water, and then partitioned successively with chloroform (ratio 1:3 *v*/*v*) and n-butanol saturated with water (ratio 1:3 *v*/*v*, three times). The n-butanol extract was combined and evaporated using a rotary evaporator at 60 °C to give a solid residue. Prior to the assay, the extracts were solubilized in 0.5 M phosphate buffer (pH 7.4). The total saponin content of each extract was determined using the method described by Xi et al. [21]. The extracts (50 µL) were mixed with 500 µL of vanillin (8% *w*/*v*) and 5 mL of sulphuric acid (72% *w*/*v*). The mixture was then incubated for 10 min at 60 °C and cooled in an ice water bath for 15 min. The absorbance was read at 538 nm. Saponin from quillaja bark was used as a reference standard, with the total saponin content of each extract expressed as g quillaja saponin equivalents (QSE)/100 g DW).

### 2.6. Determination of Condensed Tannin Content

The condensed tannins in the *T. ferdinandiana* tissues were determined using the vanillin/HCl assay described by Ahmed, et al. [22]. The extract (500 µL) was mixed with 3 mL of vanillin reagent containing 4% concentrated HCl and 0.5% vanillin in methanol in a 15 mL falcon tube. The mixture was allowed to stand for 15 min at room temperature and the absorbance was then recorded at 500 nm. Methanol was used as the blank. An appropriate standard curve was prepared using catechin, with concentrations ranging from 0.02 to 2.5 mg/mL *w*/*v*. The amount of condensed tannins in the extracts was expressed as g catechin equivalents (CaE)/100 g DW. 

### 2.7. Determination of Hydrolysable Tannin 

The hydrolysable tannins in the *T. ferdinandiana* tissue extracts were determined using the potassium iodate assay previously described by Hoang, et al. [23]. Briefly, 50 µL of 1 mg/mL *w*/*v* extract was added to a 96-well plate with 150 µL of 2.5% *w/v* potassium iodate. Absorbance was measured at 550 nm after 15 min, using a Tecan Microplate Reader (Tecan Infinite M200) with Magellan Software (version 6.4). Tannic acid was used as a standard and results were expressed as mg tannic acid equivalents (TAE)/100 g DW.

### 2.8. Antimicrobial Activity

#### 2.8.1. Foodborne Microorganisms

Foodborne microorganisms, including pathogenic and clinical isolates, were chosen for this study. A total of 4 g positive bacteria—*Staphylococcus aureus* (NCTC 6571) (National Collection of Type Cultures, Health Protection Agency Centre for Infection, London, UK), methicillin resistant *Staphylococcus aureus* (MRSA) clinical isolates (CI) (Royal Brisbane and Women’s Hospital, Herston, Queensland, AU), *Bacillus cereus* (ATCC 10876) (Microbiologics Inc., St. Cloud, MN, USA), and *Listeria monocytogenes* (ATCC 19111) (The University of Queensland, Brisbane, AU)—and 2 g negative bacteria—*Pseudomonas aeruginosa* (ATCC 10145) (Microbiologics Inc., St. Cloud, MN, USA), and *Pseudomonas aeruginosa* clinical isolates (CI) (ATCC 9001) (Royal Brisbane and Women’s Hospital, Herston, Queensland, AU)—were tested. Bacteria were maintained on plate count agar (PCA) medium at 4 °C and sub-cultured on PCA medium at 37 °C for 24 h.

#### 2.8.2. Disc Diffusion Assay

The ASE of fruits, leaves, seedcoats, and barks of *T. ferdinandiana* were diluted with 20% ethanol to prepare a final concentration 10 mg/mL *w*/*v* extract, except for the water extracts, which were diluted with reverse osmosis (RO) water. The zone of inhibition was determined using the Kirby–Bauer assay modified by Dussault, et al. [24]. The zone of inhibition was measured using a digital calliper 150 mm (ALDI, Australia). RO water and 20% ethanol were used as the negative controls. A standard antibiotic solution, oxytetracycline (0.06 mg/mL; Sigma-Aldrich, St. Louis, MN, USA), was used as positive control. All experiments were performed in triplicate and the antimicrobial activity was evaluated by measuring the inhibition zones against the tested microorganisms. 

#### 2.8.3. Determination of Minimum Inhibitory Concentration and Minimum Bactericidal Concentration 

The minimum inhibitory concentration (MIC) and minimum bactericidal concentration (MBC) of the extracts were determined by the microplate dilution method [25]. Different concentrations of the extracts were prepared using 20% ethanol and added to the microtiter wells to obtain final concentrations of 4, 3.5, 3, 2.5, 2, 1.5, 1, and 0.5 mg/mL. Nutrient broth (NB) (200 µL) was used as a control to ensure the broth was sterile, whilst 50 µL bacterial culture (1 × 10^5^ colony forming unit (CFU)/mL, as determined by colony counting after serial dilution and plating) and 150 µL nutrient broth were used as the negative control. Aliquots (100 µL) of extracts (1–8 mg/mL) were added to a 96-well microplate. A total of 50 µL bacterial culture and 50 µL NB were also added to the wells to make the final volume 200 µL. Six replicates were prepared for each concentration of the extracts and the positive antibiotic control solution (0.0625 mg/mL oxytetracycline). The microplates were incubated at 37 °C with visual observation of bacterial growth performed after 24 h. The MIC values were identified as the minimum concentration at which no visible bacterial growth was recorded [18]. The MBC was observed as the lowest concentration that completely inhibited the bacteria. A 50 µL aliquot from all wells showing no visible bacterial growth in the MIC assay [26] was applied to PCA plates and incubated at 37 °C for 24 h. The MBCs of the extracts were measured by observing the viability of the initial bacterial inoculum. 

#### 2.8.4. Scanning Electron Microscopy 

The methicillin resistant *S. aureus*, clinical isolates of *P. aeruginosa*, *L. monocytogenes*, and *B. Cereus* strains were grown for 7 h in tryptone soya yeast extract broth (TSYEB) at 37 °C. Methanolic ASE of *T. ferdinandiana* fruits and leaves were reconstituted in 75 µL 20% *v*/*v* ethanol, added to 1 mL bacteria and broth samples, and incubated for 24 h at 37 °C. The negative control was comprised of 75 µL 20% *v*/*v* ethanol. The samples and controls were washed three times in sterile phosphate buffered saline and fixed in 3% *v*/*v* glutaraldehyde [27]. Glutaraldehyde-fixed samples were fixed again in 1% *v*/*v* osmium tetroxide and dehydrated with ethanol. Samples were adhered to coverslips coated with poly-L-lysine (1 mg/mL) and dehydrated in the same manner, before being dried in a critical point dryer (Tousimis Research Corporation, Rockville, MD, USA) according to manufacturer’s instructions. Coverslips were attached to stubs with double-sided carbon tabs and coated with gold using a sputter coater (Agar Scientific Ltd, Essex, UK), following the manufacturer’s instructions. Samples were imaged in a Jeol Neoscope JCM 5000 (Jeol Ltd., Tokyo, Japan) at an accelerating voltage of 10 kV and for high resolution images in a Jeol JSM 7100F (Jeol Ltd., Tokyo, Japan) field emission scanning electron microscopy (SEM) at an accelerating voltage of 1 kV.

### 2.9. Statistical Analysis

All values were expressed as mean ± SD (*n* = 3). Statistical analysis of the results was performed using two-way ANOVA, followed by Tukey’s multiple comparison post hoc tests, with significant differences observed at *p* < 0.05 using GraphPad Prism version 8 (La Jolla, CA, USA).

## 3. Results and Discussion

### 3.1. Extraction Yields

**Figure 2:**
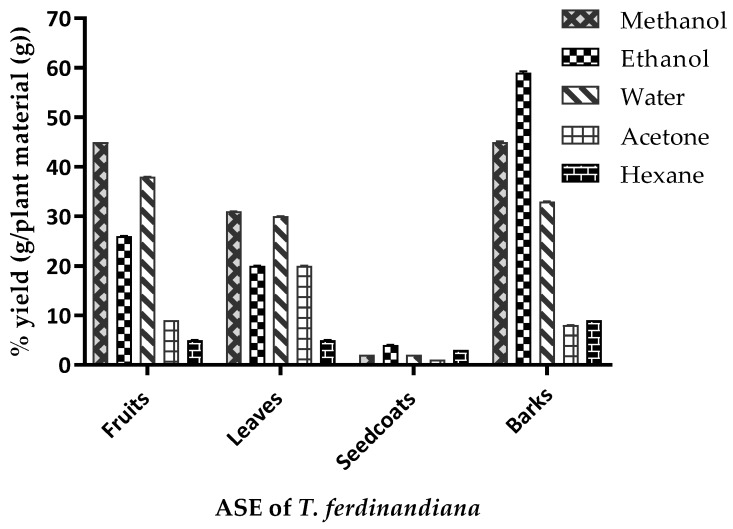
Yield (%) of the accelerated solvent extraction (ASE) of *Terminalia ferdinandiana*. Results are shown as the mean of triplicate experiments ± SD.

The ASE yields from the *T. ferdinandiana tissues* are presented in Figure 2 and vary depending upon the plant tissue and solvent used. The yield variations observed from the same tissues following different ASE methods could be attributed to the varying polarities of compounds present in the various tissues [1]. A yield of 45% was achieved from fruit and bark powders using methanol extraction, whilst ethanol produced extract yields of 59% and 26% from barks and fruit powders, respectively. Water extraction produced similar yields from fruits, leaves, and barks, ranging from 30–40%. Acetone and hexane produced comparatively lower extract yields to other solvents, except from leaves, where acetone produced an extract yield similar to ethanol. The variable yields achieved by the different ASE methods could be due to the solubility of the phytochemicals in the different tissues, as well as the duration, temperature, and pH of the extraction conditions, along with the particle size of the sample and the solvent-to-sample ratio [28].

### 3.2. Antioxidant Capacity

#### 3.2.1. Total Phenolic Content 

The total phenolic contents (TPC) of all extracts are presented in Table 1 and ranged from 0.04–24 g GAE/100 g DW. In the *T. ferdinandiana* fruits extracts, TPC ranged from 0.38–12 g/100 g DW. The barks and leaves also contained high TPC, ranging from 0.04–2 g/100 g DW. The highest TPC was measured in methanol ASE from the fruit and leaves, followed by ethanol > acetone > water > hexane. In the bark ASE, ethanol produced the highest TPC, followed by methanol > water > acetone > hexane. The seedcoat extracts produced the lowest TPC overall, however had similar trends to the bark extracts, with ethanol > methanol ≈ water > acetone > hexane. 

The variation in TPC observed in the different extracts could be due to the variable solubility of polyphenols in different solvents, as well as the complex structure of the cellular macromolecules within different plant tissues. The enrichment of phenolic compounds in the extracts also depended on the solvent and the process of extraction [29]. For example, it is possible that hexane primarily extracted non-polar components, such as chlorophyll, waxes, and terpenoids from the tissues producing the lowest TPC. Regardless of the method used to prepare extracts from the different *T. ferdinandiana* tissues, the TPC content in the extracts indicates enrichment with phenolic compounds, which are potent scavengers of free radicals in vitro, and are believed to provide in vivo antioxidant protection against biomolecule damage and peroxidation of cellular membranes [30].

#### 3.2.2. DPPH radical Scavenging Capacity 

2, 2-diphenyl-1-picrylhydrazyl (DPPH) is a stable free radical widely accepted as a tool for estimating the free radical-scavenging capacity of an antioxidant. The effect of an antioxidant on DPPH radical scavenging is determined by the ability of the antioxidant to donate hydrogen [31]. The ASE extracts of *T. ferdinandiana* tissues were assayed for DPPH radical scavenging capacity. Results are presented in Table 2, showing that ASE extracts from the different tissues produced similar results, except for hexane ASE, which showed very low radical scavenging activity. Plant antioxidants are mostly water-soluble and present as glycosides located in the cell vacuole [32]. This is consistent with the findings in Table 2, where polar solvents produced extracts with greater antioxidant potential than non-polar solvents. 

### 3.3. Determination of Total Saponins

Saponins are bitter tasting, water-soluble triterpenoids found in various plants. Saponins have been found to possess in vitro anti-inflammatory activities [33], however the bitter taste may limit applications when present in higher quantities. The increased consumer demand for natural products with beneficial physicochemical and biological properties makes steroidal and triterpenoid saponins promising compounds for industrial applications [34]. The major sugar moieties of saponins are glucose, arabinose, galactose, glucuronic acid, xylose, and rhamnose [35]. Saponin-containing plants used for human consumption include soybeans, pulses, peas, chickpea, lentils, oats, potatoes, pepper, tomatoes, onions, garlic, tea, asparagus, cucumber, pumpkins, squash, gourds, melons, watermelons, sugar beet, yam, sunflower, and cassava [36,37]. The saponin content of lentils ranges from 3.7 to 4.6 g/kg, green peas 11 g/kg, chickpeas 60 g/kg, oats 1 g/kg and spinach 47 g/kg DW [37]. The saponin contents of *T. ferdinandiana* tissues are presented in Table 3. The slightly bitter taste of *T. ferdinandiana* fruits might be due to the presence of low amounts of saponins. Barks were highest in saponins, whereas no saponins could be detected in the seedcoats. 

### 3.4. Determination of Condensed and Hydrolysable Tannins

Tannins are higher molecular weight polyphenolics, found mostly in plants used as food and feed [38]. Tannins are usually divided into two groups—hydrolysable and condensed tannins. The degree of tannin polymerization has been found to directly correlate with radical scavenging capacity [39,40]. The antioxidant effects of tannins are mostly attributed to free radical scavenging capacity, chelation of transition metals, inhibition of pro-oxidative enzymes, and lipid peroxidation [39]. Tannins have varying degrees of hydroxylation, and their molecular size is sufficient to form complexes with proteins [41].

Condensed tannin contents of *T. ferdinandiana* tissues are presented in Table 3, showing that bark has the highest content compared to leaves, which have the lowest content. Fruits were found to contain 0.8 g condensed tannins/100 g DW. The hydrolysable tannin contents of *T. ferdinandiana* tissues are shown in Table 4. The hydrolysable tannin content of the tissues ranges between 0.1 and 120 mg/100 g DW of the plant material. Leaves contain more tannins than fruits for all solvent extracts. Very low levels of tannins were observed in all seedcoat extracts, except for the acetone ASE. The highest amount of hydrolysable tannins was found in leaves (Table 4). These results suggest that the level of hydrolysable tannins is greatly influenced by the tissue type, solvents (different polarities), and extraction conditions (Table 4). The results of the present study indicate that tannins in *T. ferdinandiana* fruit extracts were predominantly condensed tannins.

Using regression analyses, the correlations between TPC, DPPH radical scavenging values, saponin content, and the condensed and hydrolysable tannin content of the fruits, leaves, seedcoats, and barks were explored. The antioxidant capacity and tannin content of the fruits were positively correlated with the antioxidant capacity and tannin content of the leaves (pearson *R^2^* = 0.9736, *p* < 0.0001), seedcoats (pearson *R^2^* = 0.6886, *p =* 0.0001), and barks (pearson *R^2^* = 0.7728, *p* < 0.0001). The saponins content of the tissues was positively correlated with the condensed tannins (pearson *R^2^*=0.9922, *p* < 0.05).

### 3.5. Antimicrobial Activity

#### 3.5.1. Disc Diffusion Assay

**Figure 3:**
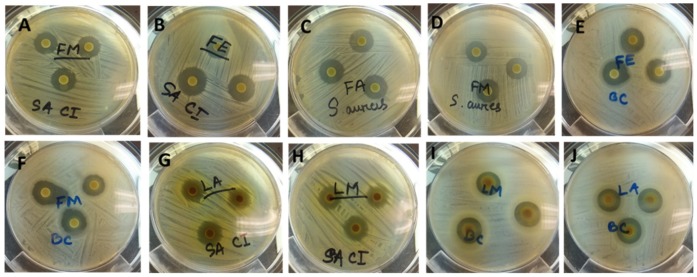
Antimicrobial activity (zones of inhibition) of ASE extracts of *T. ferdinandiana* tissues against various organisms. FM and FE against MRSA (A and B), FA and FM against *Staphylococcus aureus* (C and D), FE and FM against *Bacillus cereus* (E and F), LA and LM against MRSA (G and H), LM and LA against *B. cereus* (I and J). FM; Fruit methanol extract, FE; Fruit ethanol extract, FA; Fruit acetone extract, LA; Leaf acetone extract, LM; Leaf methanol extract; MRSA; Methicillin resistant *Staphylococcus aureus.*

Plant derived antimicrobials can effectively reduce or inhibit pathogenic and spoilage microorganisms and have the potential to be an alternative to synthetic antimicrobials [6]. The use of natural antimicrobial agents in food processing to extend the shelf-life of food products is well documented [6]. Consumer concern over synthetic preservatives in food products has contributed to the search for preservatives from natural sources. The antimicrobial activities of extracts from *T. ferdinandiana* tissues prepared with different solvents were determined against different microorganisms, with the inhibition zone measured in mm, and presented in Table 5 and illustrated in Figure 3. Overall, methanol extracts were found to be the most effective against the organisms tested and showed a broad spectrum of antimicrobial activity against the tested bacteria. The antimicrobial activity of the methanol extracts was similar to the acetone extracts, whilst water extracts from fruit, leaves, and bark were found to be active against *S. aureus*, MRSA, *P. aeruginosa* CI, and *B. cereus.* Fruit and leaf extracts were found to have similar zones of inhibition against the tested organisms, with MRSA, *L. monocytogenes,* and *B. cereus* the most sensitive bacteria among those tested. *S. aureus* was inhibited less compared to MRSA. Seedcoat extracts were found to be the least active against the microorganisms tested.

Herbal remedies formulated from whole plants are gaining more interest, as they are safer than synthetic options. The antimicrobial activity from *T. ferdinandiana* extracts against different microbial strains supports the scientific rationality of using plants/plant tissue in traditional medicine [42]. The inhibition of the growth of six bacterial strains by the fruit and leaf extracts could be due to the presence of antioxidant phytochemicals, mainly polyphenols, in the extracts. The *T. ferdinandiana* results support several other studies, showing the antimicrobial activity of plant extracts due to the presence of polyphenolic compounds in the extracts [26,43]. Polyphenols, particularly tannins and flavonols, are known to possess antimicrobial activity and can suppress the growth of microorganisms by various mechanisms, such as the inhibition of biofilm formation, host-ligand adhesion reduction, and the neutralization of bacterial toxins [44].

In the present study, we found that *T. ferdinandiana* tissue extracts are high in TPC and tannins. Other species of Terminalia plants, such as *Terminalia arjuna*, *Terminalia bellerica*, *Terminalia chebula*, *Terminalia sambesiaca*, *Terminalia Kaiserana* and *Terminalia sericia,* are also high in tannins and other polyphenols [45,46,47]. Previous reports on the antimicrobial properties of Terminalia plants were supported by the presence of a vast range of phytochemicals, including polyphenols and tannins [48,49,50]. Tannins inhibit bacterial growth by binding to bacterial enzymes and interfering with phosphorylation, and sometimes forming complexes with transition metal ions, which are important for bacterial growth [51].

#### 3.5.2. Determination of Minimum Inhibitory Concentration (MIC) and Minimum Bactericidal Concentration (MBC)

MIC and MBC values of the extracts of *T. ferdinandiana* tissues against the tested microbial strains are shown in Table 6. In general, the MIC and MBC values of the extracts against the tested microorganisms ranged from 1.0 mg extract/mL to 3.0 mg/mL, with *L. monocytogenes*, *B. cereus,* and MRSA the most sensitive among the tested microorganisms. Different tissues extracted with different solvents showed variable inhibitory effects. For example, the MIC of *S. aureus* was 1 mg/mL against bark ethanol extracts, whereas the MIC in response to bark water extracts was 3 mg/mL. The MIC of *P. aeruginosa*, *P. aeruginosa* CI, *L. monocytogenes,* and *B. cereus* ranged from 1 to 2 mg/mL of extracts, however for *S. aureus* and MRSA, 3 mg/mL was needed for inhibition.

Overall, the ethanol and acetone extracts were the most effective at inhibiting the growth of the microorganisms compared to methanol and water extracts. It is interesting to note that even though the antioxidant capacity and phenolic content of the methanol and water extracts was found to be higher than the ethanol extracts, the acetone extracts showed lower antioxidant capacity and phenolic contents overall. The phytochemicals responsible for antioxidant capacity may not be the only compounds contributing to antimicrobial activity, it is possible that other phytochemicals with antimicrobial potentials are exerting antimicrobial activities.

#### 3.5.3. Scanning Electron Microscopy 

**Figure 4:**
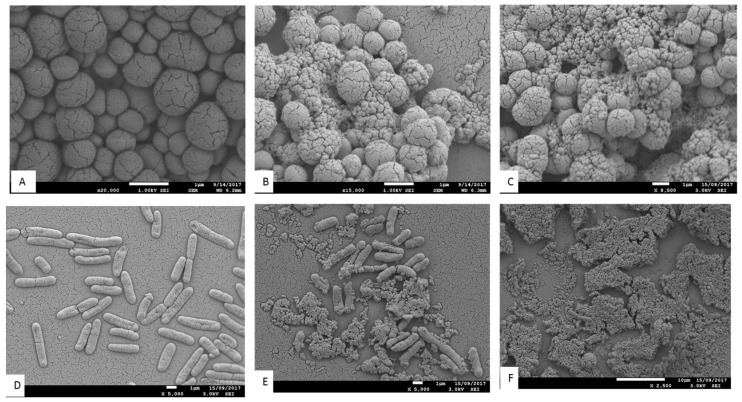
Antimicrobial activity of methanolic ASE of *T. ferdinandiana* fruits and leaves. Scanning electron microscopy (SEM) images of methicillin resistant *Staphylococcus aureus* control (**A**), effect of fruit extracts (**B**), effect of leaf extracts (**C**), and clinical isolates of *Pseudomonas aeruginosa* control (**D**), effect of fruit extracts (**E**) and effect of leaf extracts (**F**). Samples were imaged in a Jeol JSM 7100F field emission SEM at an accelerating voltage of 1 kV.

**Figure 5:**
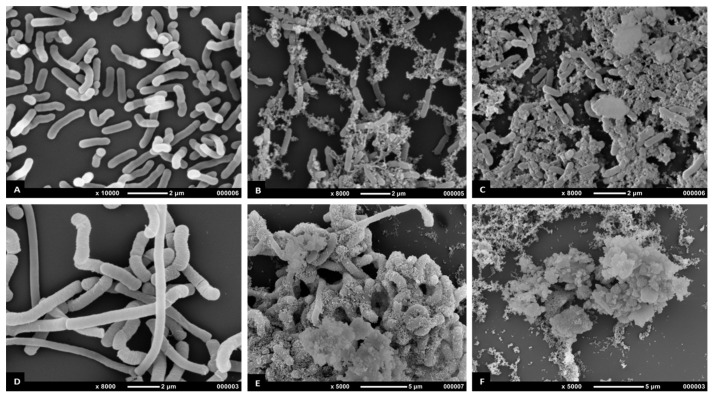
Antimicrobial activity of methanolic ASE of *T. ferdinandiana* fruits and leaves. SEM images of *Listeria monocytogenes* treated with control (**A**), fruit extracts (**B**), and leaf extracts (**C**), and *Bacillus cereus* treated with control (**D**), fruit extracts (**E**), and leaf extracts (**F**). Samples were imaged in a Jeol Neoscope JCM 5000 at an accelerating voltage of 10 kV.

The antimicrobial effects of *T. ferdinandiana* fruit and leaf extracts on the morphology of MRSA, *B. cereus*, *L. monocytogenes,* and *P. aeruginosa* CI cells were determined by scanning electron microscopy, as illustrated in Figure 4 and Figure 5. All bacterial cells treated with the extracts at the MIC were damaged compared to the control cells (20% *v*/*v* ethanol). The control cells had a smooth surface, with the outer layer of the bacteria relatively intact (Figure 4A,D and Figure 5A,D). By contrast, the damaging effects of the fruit and leaf extracts on bacterial cell walls were evident compared to the appearance of the control cells (Figure 4B,C,E,F and Figure 5B,C,E,F). Almost all the bacterial cells treated with the fruit and leaf extracts showed the disintegration of the outermost layer and, in some cases, the outermost layer had disappeared (Figure 4F and Figure 5E,F).

The antimicrobial mechanisms or exact target sites for natural antimicrobials have not been identified yet and warrant further investigation [6]. However, it is thought that terpenoids and phenolics are involved in membrane disruption, phenolic acids and flavonoids cause metal chelation, coumarin interferes with the genetic material, and alkaloids inhibit the growth of microorganisms [52]. Phytochemicals are also reported to be involved in membrane disruption and, in turn, cause leakage of cellular content [53]. It was observed that plant phytochemicals interfere with active transport mechanisms and possibly dissipate cellular energy in adenosine triphosphate (ATP) form [54]. 

In Figure 5B,C, some of the extract-treated *L. monocytogenes* cells underwent splitting, a change in cell morphology due to deep wrinkling and distortion. Therefore, it is postulated that fruit and leave methanol extracts have antimicrobial activity against *L. monocytogenes*. Antioxidative polyphenols might have been involved in causing lesions in the cytoplasmic membrane, which in turn may have caused leakage of intracellular contents, impairment of microbial enzymes, and potentially cell death [53]. This evidence suggests that *T. ferdinandiana* fruits extracts may effectively inhibit *L. monocytogenes* in food products.

To visualize the effects of *T. ferdinandiana* fruit and leaf methanol extracts, SEM images of *B. cereus* cells treated with MIC doses of extracts were taken and are presented in Figure 5. The fruit and leaf extracts altered the cell morphology (Figure 5E,F) in comparison to controls (Figure 5D). The control bacterial cells appeared whole and distinct from one another, whilst the bacterial cells treated with both the fruit and leaf extracts were deformed. In particular, the cell wall of *B. cereus* treated with leaf extracts appeared to be degraded (Figure 5F). 

A change in cell morphology was observed in *P. aeruginosa* clinical isolates incubated with *T. ferdinandiana* fruit and leaf extracts, as shown in Figure 4E and 4F. The cell surface morphology of *P. aeruginosa* control cells was intact and smooth (Figure 4D) compared to cells incubated with the MIC of *T. ferdinandiana* fruit extracts, which changed to granular with the appearance of blisters (Figure 4E). Treatment with leaf extracts was even more pronounced, as evidenced by the loss of cellular orientation (Figure 4F). These results suggest that *T. ferdinandiana* leaf extracts are more active than fruit extracts in promoting *P. aeruginosa* cell death caused by cell membrane disintegration and cell atrophy, indicating that the active compounds present in *T. ferdinandiana* leaf extracts may act on the cell membrane or extracellular proteins, resulting in the inhibition of bacterial cell growth.

Scanning electron microscopy images of MRSA (Figure 4B,C) treated with *T. ferdinandiana* extracts also showed partial disintegration of the bacterial cell surfaces and reduced residual cellular content. Cell surfaces also appeared rougher after *T. ferdinandiana* extract treatment. The potent antimicrobial activity observed in the *T. ferdinandiana* extracts in the present study can therefore be attributed to the presence of numerous phytochemicals in the plant, especially ascorbic and ellagic acid, as previously reported [15]. In the presence of *T. ferdinandiana* extracts, bacterial cells grew as isolated colonies, compared to control cells. The antimicrobial activity of plants is mostly attributed to their principal phenolic components, which exhibit significant bactericidal activity against MRSA. A reaction between phenolic compounds and bacterial membrane proteins was suggested to be involved in their antimicrobial action, which can weaken the cell wall or damage the cytoplasmic membrane directly [55].

These results indicate that antimicrobial compounds are contained in *T. ferdinandiana* leaves and fruit and act by damaging bacterial cell walls or inducing cell lysis. It is possible that the antimicrobial compounds present in *T. ferdinandiana* extracts readily enter the cells through these lesions, whilst also facilitating the leakage of cell contents. That is, when microbial cell walls or membranes become compromised, possibly by interacting with phenolic compounds, low molecular weight substances, such as K^+^ and PO_4_^3−^, tend to leach out first, followed by the loss of other intracellular molecules, such as proteins, DNA, RNA, and other higher molecular weight materials [56]. These antimicrobial compounds may even react with bacterial DNA, ultimately resulting in cell death. Some researchers have reported that bioactive compounds derived from plants have antimicrobial effects on cells through reduced oxygen uptake, reduced cellular growth, inhibition of lipid, protein, and nucleic acid synthesis, changes in the lipid profile of the cell membrane, and inhibition of microbial cell wall synthesis. Cox et al. [57] reported that slight changes in the structural integrity of cell membranes can affect cell metabolism and lead to cell death. 

A wide variety of phenolic compounds, including tannins, gallic acid, ellagic acid, corilagin, geraniin, tannic acid, punicalagin, castalagin, and punicalin, have been reported to be present in the *Terminalia* genus [58]. Antimicrobial activity of these compounds has also been reported against a number of microorganisms, such as MRSA, *S. aureus*, *P. aeruginosa*, Genus *vibrio*, *Escherichia coli*, *Candida Albicans*, and *Aspergillus fumigatus* [59]. Previous reports on the phytochemicals present in *T. ferdinandiana* include gallic acid, apionic acid, gluconolactone, chebulic acid, ferulic acid, exifone, corilagin, punicalin, castalagin, and chebulagic acid [14,60]. High levels of ellagic acid and ascorbic acid have also been reported in *T. ferdinandiana* [15]. *T. ferdinandiana* fruit is currently marketed commercially as a functional ingredient in the form of a freeze-dried powder in the food industry, however, other tissues such as leaves have not yet been considered as functional (food) ingredients. 

## 4. Conclusions

The contamination of food by microorganisms is a worldwide public health problem. To avoid these problems, plant-derived natural preservatives could offer a safer alternative. To date, this is the first study to extensively investigate the antimicrobial properties of *T. ferdinandiana* extracts, revealing that extracts of *T. ferdinandiana* fruit and leaves possess significant in vitro antimicrobial properties against common foodborne bacteria. The antimicrobial properties of this plant were also supported by the presence of significant antioxidant and tannin contents. Overall, the results of our present study showed that *T. ferdinandiana* fruit and leaves have great potential as natural preservatives in the food industry. However, further research on the bioactive compounds present in *T. ferdinandiana* extracts is needed to determine the compounds responsible for the antimicrobial properties.

## Figures and Tables

**Table 1:** Total phenolic contents in *T. ferdinandiana* tissues.

	Total Phenolic Content (GAE g/100 g DW)			
	Fruits	Leaves	Seedcoats	Barks
Methanol	12.2 ± 2.9 ^a, w^	11.7 ± 0.5 ^a, w^	0.2 ± 0.0 ^x^	18.0 ± 2.0 ^a, y^
Ethanol	11.6 ± 1.0 ^a, w^	8.8 ± 0.5 ^b, x^	0.3 ± 0.0 ^y^	23.5 ± 0.5 ^b, z^
Water	5.2 ± 0.2 ^b, w^	4.2 ± 0.4 ^b, c, x^	0.2 ± 0.0 ^y^	6.7 ± 0.2 ^c, w^
Acetone	8.0 ± 0.2 ^b, w^	5.2 ± 0.2 ^c, w^	0.1 ± 0.0 ^x^	3.5 ± 0.0 ^d, z^
Hexane	0.4 ± 0.0 ^c^	0.2 ± 0.0 ^d^	ND	0.04 ± 0.0 ^e^

Results are expressed as mean ± SD; (*n* = 3). Mean values of each column with different letters are significantly different (at *p* < 0.05). a, b, c, d, e; denote significant differences of extraction solvents within same tissue. w, x, y, z; denote significant differences of the same extraction solvent across tissues.

**Table 2:** The 2, 2-diphenyl-1-picrylhydrazyl (DPPH) radical scavenging capacity of *T. ferdinandiana* tissues.

	DPPH Radical Scavenging Activity (%)			
Accelerated solvent extracts (ASE) of *T. ferdinandiana*	Fruits	Leaves	Seedcoats	Barks
Methanol	93.4 ± 0.3 ^a, x^	89.4 ± 0.5 ^a, x^	93.0 ± 0.2 ^a, x^	84.7 ± 0.1 ^a, y^
Ethanol	94.3 ± 0.1 ^a,x^	91.8 ± 0.2 ^a,y^	88.0 ± 0.2 ^b,y^	85.8 ± 0.2 ^a,z^
Water	93.7 ± 0.2 ^a, x^	84.0 ± 0.5 ^b, y^	90.9 ± 0.2 ^b, x^	79.6 ± 0.2 ^b, y^
Acetone	91.5 ± 0.4 ^a, w^	79.2 ± 0.3 ^c, x^	74.2 ± 0.3 ^c, x^	85.5 ± 0.3 ^a, y^
Hexane	12.9 ± 0.9 ^b, w^	68.7 ± 0.4 ^d, x^	2.1 ± 1.3 ^d, y^	77.5 ± 3.8 ^b, z^

Results are expressed as mean ± SD; (*n* = 3). Mean values of each column with different letters are significantly different (at *p* < 0.05). a, b, c, d; denote significant differences of extraction solvents within same tissue. w, x, y, z; denote significant differences of the same extraction solvent across tissues.

**Table 3:** Saponins and condensed tannins in *T. ferdinandiana* tissues.

*T. ferdinandiana* Tissues	Saponin Content (QSE g/100 g DW)	Condensed Tannin Content (CaE g/100 g DW)
**Fruits**	0.4 ^a^ ± 0.0	0.8 ^a^ ± 0.1
**Leaves**	0.3 ± 0.1	0.02 ^b^ ± 0.0
**Seedcoats**	ND	0.1 ^a^ ± 0.0
**Barks**	7.0 ^b^ ± 0.2	7.0 ^c^ ± 0.7

Results are expressed as mean ± SD; (*n* = 3). ND = not detected. Mean values of each column with different letters are significantly different (*p* < 0.05).

**Table 4:** Hydrolysable tannins in *T. ferdinandiana* tissues.

	Hydrolysable Tannin Content (TAE mg/100 g DW)			
ASE	Fruits	Leaves	Seedcoats	Barks
**Methanol**	55.3 ± 1.6 ^a,w^	120.8 ± 2.3 ^a, x^	0.9 ± 0.0 ^a, y^	16.5 ± 0.2 ^a, z^
**Ethanol**	33.3 ± 0.8 ^b, w^	81.4 ± 1.4 ^b, x^	1.42 ± 0.0 ^a, y^	20.4 ± 0.2 ^b, z^
**Water**	7.5 ± 0.4 ^c, w^	52.0 ± 0.5 ^c, x^	1.42 ± 0.1 ^a, y^	13.6 ± 0.0 ^c, z^
**Acetone**	10.8 ± 0.7 ^d, w^	66.5 ± 1.1 ^d, x^	27.1 ± 1.8 ^b, y^	4.9 ± 0.0 ^d, z^
**Hexane**	0.1 ± 0.1 ^e, w^	3.1 ± 0.2 ^e, x^	2.6 ± 0.2 ^a, x^	0.2 ± 0.4 ^e, w^

Results are expressed as mean ± SD; (*n* = 3). Mean values of each column with different letters are significantly different (*p* < 0.05). a, b, c, d, e; denote significant differences of extraction solvents within same tissue. w, x, y, z; denote significant differences of the same extraction solvent across tissues.

**Table 5:** Antimicrobial activity of the extracts of *T. ferdinandiana* tissues.

ASE Extraction Solvent	*T. ferdinandiana* Tissues	Zone of Inhibition (in mm)					
		*S. aureus*	MRSA	*Pseudomonas aeruginosa*	*P. aeruginosa* (CI)	*B. cereus*	*Listeria monocytogenes*
**Methanol**	**Fruits**	13.8 ± 0.3 ^a, w^	16.4 ± 0.0 ^a, x^	--	11.2 ± 0.0 ^a, w^	16.4 ± 0.9 ^a, x^	20.4 ± 2.0 ^a, y^
	**Leaves**	--	15.2 ± 0.4 ^a, w^	--	14.6 ± 1.5 ^b, w^	16.0 ± 0.6 ^a,w^	21.3 ± 0.2 ^a, x^
	**Seedcoats**	--	8.8 ± 0.0 ^b, w^	--	--	11.5 ± 0.6 ^b, x^	11.4 ± 0.8 ^b, x^
	**Barks**	11.6 ± 0.4 ^a^	12.0 ± 0.8 ^c^	--	--	12.8 ± 0.3 ^b^	--
**Water**	**Fruits**	--	--	--	12.9 ± 1.4 ^a^	--	--
	**Leaves**	--	13.3 ± 1.4 ^c, w^	--	10.7 ± 0.8 ^a, x^	--	--
	**Seedcoats**	--	--	--	--	--	--
	**Barks**	10.8 ± 1.4 ^a^	11.6 ± 0.5 ^c^	--	--	11.1 ± 0.4 ^b^	--
**Ethanol**	**Fruits**	--	17.1 ± 0.1 ^a^	--	--	17.8 ± 0.6 ^a^	18.5 ± 0.5 ^a^
	**Leaves**	--	14.6 ± 0.2 ^a, w^	--	--	16.5 ± 1.0 ^a, x^	20.0 ± 0.6 ^a, y^
	**Seedcoats**	--	--	--	--	9.8 ± 0.8 ^c^	10.8 ± 0.6 ^b^
	**Barks**	12.1 ± 0.3 ^a^	12.7 ± 0.9^c^	--	--	13.2 ± 1.1 ^b^	--
**Acetone**	**Fruits**	16.7 ± 0.5 ^b, w^	16.6 ± 0.3 ^a, w^	--	13.3 ± 0.1 ^b, x^	18.4 ± 1.5 ^a, y^	20.5 ± 0.4 ^a, z^
	**Leaves**	--	15.7 ± 0.9 ^a, w^	8.7 ± 0.8 ^a, x^	14.1 ± 0.1 ^b, w^	16.1 ± 0.3 ^a, w^	21.0 ± 1.3 ^a, y^
	**Seedcoats**	--	--	--	--	--	--
	**Barks**	15.0 ± 1.0 ^b, w^	11.0 ± 1.5 ^c, x^	--	--	15.7 ± 0.6 ^a, w^	15.3 ± 1.8 ^c, w^
**Oxytetracycline (0.25 mg/mL)**		33.9 ± 0.0 ^c, w^	29.7 ± 1.9 ^d, w^	13.8 ± 0.5 ^b, x^	18.1 ± 0.6 ^c, y^	17.3 ± 1.5 ^a, y^	33.3 ± 0.4 ^d, w^

Results are expressed as mean ± SD; (*n* = 3). Mean values of each column are significantly different (*p* < 0.05). (--) denotes that no zone of inhibition was observed. Criteria for antimicrobial activity: <10 mm = weak, 10–15 mm = moderate, and >15 mm = strong. Mean values of each column with different letters are significantly different (*p* < 0.05). a, b, c, d; denote significant differences of extraction solvents within same tissue. w, x, y, z; denote significant differences of the same extraction solvent across tissues. Controls (reverse osmosis (RO) water and 20% ethanol) did not show any zone of inhibition.

**Table 6:** The minimum inhibitory concentration (MIC) and minimum bactericidal concentration (MBC) of the extracts of *T. ferdinandiana* tissues.

**Tested Microorganisms**	**MIC (mg/mL)**													
	**FM**	**LM**	**SM**	**BM**	**FW**	**LW**	**BW**	**FE**	**LE**	**SE**	**BE**	**FA**	**LA**	**BA**
***Staphylococcus aureus***	1.5			2			3				1	1		2
**MRSA**	3	2.5		1			2.5	3	3		3	3		1
***Pseudomonas aeruginosa***	1				1	1			1			1		
***Pseudomonas aeruginosa* CI**						1			1			1		
***Bacillus cereus***	1.5	1	1	1.5			1	1	1	1	1		1	1
***Listeria monocytogenes***	1	1	1					1	2	1		1		1
	**MBC (mg/mL)**													
	**FM**	**LM**	**SM**	**BM**	**FW**	**LW**	**BW**	**FE**	**LE**	**SE**	**BE**	**FA**	**LA**	**BA**
***Staphylococcus aureus***	1.5			1			2				1	1		2
**MRSA**	3	3		1			2	2	3		2	3		1
***Pseudomonas aeruginosa***	1				1	1			1			1		
***Pseudomonas aeruginosa* CI**						1			1			1		
***Bacillus cereus***	1.5	1	1	1				1	1	1	1		1	1
***Listeria monocytogenes***	1	1	1					1	2	1		1		1

MIC and MBC values ranges between 1–3 mg/mL. CI—clinical isolates, FM—fruit methanol, LM—leaf methanol, SM—seedcoat methanol, BM—bark methanol, FW—fruit water, LW—leaf water, SW—seedcoat water, BW—bark water, FE—fruit ethanol, LE—leaf ethanol, SE—seedcoat ethanol, BE—bark ethanol, FA—fruit acetone, LA—leaf acetone, BA—bark acetone. Controls did not inhibit the growth of any of the tested microorganisms.
